# Beyond the Lungs: An Atypical Presentation of Renal Tuberculosis

**DOI:** 10.7759/cureus.77272

**Published:** 2025-01-11

**Authors:** Mariana Nunes, Natacha Mourão, Beatriz R Marques, Mariana Certal, Cristiana Pinto

**Affiliations:** 1 Internal Medicine, Centro Hospitalar de Trás-os-Montes e Alto Douro, Vila Real, PRT

**Keywords:** co-infection, cystic renal lesion, extrapulmonary tuberculosis, immunocompromised patient, mycobacterium tuberculosis, renal tuberculosis

## Abstract

Renal tuberculosis (TB), an underrecognized form of extrapulmonary TB, often presents with nonspecific symptoms and challenging diagnosis and, therefore, it is difficult to diagnose. The subtle clinical manifestations and the absence of typical pulmonary signs frequently delay recognition and treatment. A 66-year-old man with type 2 diabetes mellitus and a cortical cyst in the left kidney presented to the emergency room with fatigue, anorexia, weight loss, and intermittent fever. Image exams revealed a large left renal cystic lesion initially suggestive of a cystic nephroma, which is a benign lesion. Ultrasound-guided aspiration and drainage of the lesion, followed by microbiological evaluation, identified co-infection with *Mycobacterium tuberculosis* and *Escherichia coli*. The patient received targeted antibacterial therapy with ceftriaxone and standard anti-tubercular therapy (isoniazid, rifampicin, pyrazinamide, and ethambutol) after pulmonary TB was excluded. His condition improved, and follow-up imaging showed a reduction of the cystic mass. Two weeks later, the pigtail, inserted at the time of the drainage of the lesion, was removed, and the patient was discharged with a referral to a Pulmonary Diagnostic Center for the continuation of TB treatment. This case highlights the diagnostic complexity of renal TB and the importance of considering it in the differential diagnosis of atypical renal lesions, particularly in patients with risk factors. Early intervention, accurate microbiological diagnosis, and appropriate anti-tubercular therapy can lead to favorable outcomes.

## Introduction

Tuberculosis (TB) remains a significant global health issue, with an estimated 10.8 million new cases and 1.25 million deaths worldwide in 2023, according to the World Health Organization (WHO) [1]. While pulmonary TB constitutes most cases, extrapulmonary forms are being increasingly recognized, accounting for up to 15% of all TB cases in immunocompetent individuals and an even higher proportion among those who are immunocompromised [1,2]. Among extrapulmonary manifestations, genitourinary TB ranks as one of the most common, encompassing both renal parenchymal and lower urinary tract involvement [3].

Renal TB, a subset of genitourinary TB, emerges from hematogenous dissemination, often during primary infection or reactivation of latent disease [3]. However, it is estimated that the latency period between pulmonary infection and the appearance of the first urinary symptoms is about 22 years, and only 36.5% of patients have a history of TB or evidence on imaging tests [2]. The clinical manifestations can be nonspecific, ranging from asymptomatic microscopic hematuria to advanced kidney destruction and chronic kidney disease [4,5]. Such subtle presentations and the prolonged latency period between initial infection and clinical disease onset lead to diagnostic challenges, often with delayed recognition and treatment.

Here, we present a clinical case of renal TB, highlighting the diagnostic journey, therapeutic considerations, and implications for patient care. Our case emphasizes the need for timely, accurate diagnosis and effective management strategies to prevent adverse outcomes. In an era where TB control remains a global priority, increased awareness of extrapulmonary manifestation of TB, including renal TB, can enhance clinician vigilance and improve patient prognosis.

## Case presentation

A 66-year-old Caucasian man with a known medical history of type 2 diabetes mellitus and a cortical cyst in the left kidney presented to the emergency department with pronounced fatigue, anorexia, weight loss, and abdominal distension for the past 15 days. The previous four days, he experienced intermittent fever reaching up to 39°C and night sweats. He reported no respiratory, cardiac, genitourinary, or neurological symptoms. The patient denied knowledge of contact with TB and also denied traveling abroad. There was no history of substance use.

On physical examination, the patient appeared drowsy and sluggish but was arousable with minimal stimulation and was oriented to time, place, and person. There were no signs of jaundice. Normal vital signs, except for slight tachycardia (105 beats per minute). The cardiac auscultation was normal, and pulmonary auscultation revealed a few crackles on the left side. The abdomen was soft and distended, with no evidence of ascites. No peripheral edema.

Initial laboratory tests demonstrated elevated inflammatory markers, while urine analysis was unremarkable. HIV testing was negative. The initial blood findings are shown in Table 1. 

**Table 1:** Analytic study at admission aPTT: activated partial thromboplastin time; ALT: alanine transaminase; AST: aspartate transaminase; INR: international normalised ratio; MCV: mean corpuscular volume; γ-GT: gamma-glutamyl transferase; HBV: hepatitis B virus; HCV: hepatitis C virus; HIV: human immunodeficient virus.

Parameter	Result	Reference Value
Blood analysis		
Hemoglobin	11.5 g/dL	12-16 g/dL
MCV	85 fL	87-103 fL
Leucocyte count	9400	4-11 x 10^3^/uL
Platelets	337000	150-400 x 10^3^/uL
Urea/creatinine	41/1.10 mg/dL	<50/0.5-0.9 mg/dL
Sodium/Potassium	133/4.0 mEq/L	135-147/3.7-5.1 mEq/L
AST/ALT	16/5 U/L	<35 U/L/<33 U/L
γ-GT	10 U/L	7-32 U/L
Alkaline Phosphatase	55 U/L	35-105 U/L
Total Bilirubin	0.4 mg/dL	<1.2 mg/dL
C Reactive Protein	21.90 mg/dL	<0.5 mg/dL
INR	1.21	<1.2
aPTT	33.3 seg	27-38 seg
HIV antibody	Non-reactive	
HBV antibodies	Non-reactive	
HBV HBs antigen	Non-reactive	
HCV antibody	Non-reactive	
Urine analysis		
Macroscopic examination		
Clarity	Clear	
Color	Amber	
Specific gravity	1.037	1.002-1.030
pH	5.5	
Glucose	Negative	
Ketone bodies	Negative	
Bilirubin	Negative	
Nitrites	Negative	
Protein	30.0 mg/dL	<30.0 mg/dL
White blood cells	1+	
Blood	Negative	
Microscopic examination		
Squamous epithelial cells	5-10 /field	
White blood cells	0-2 /field	
Red blood cells	0-2 /field	
Casts	0-2 /field	

**Figure 1:**
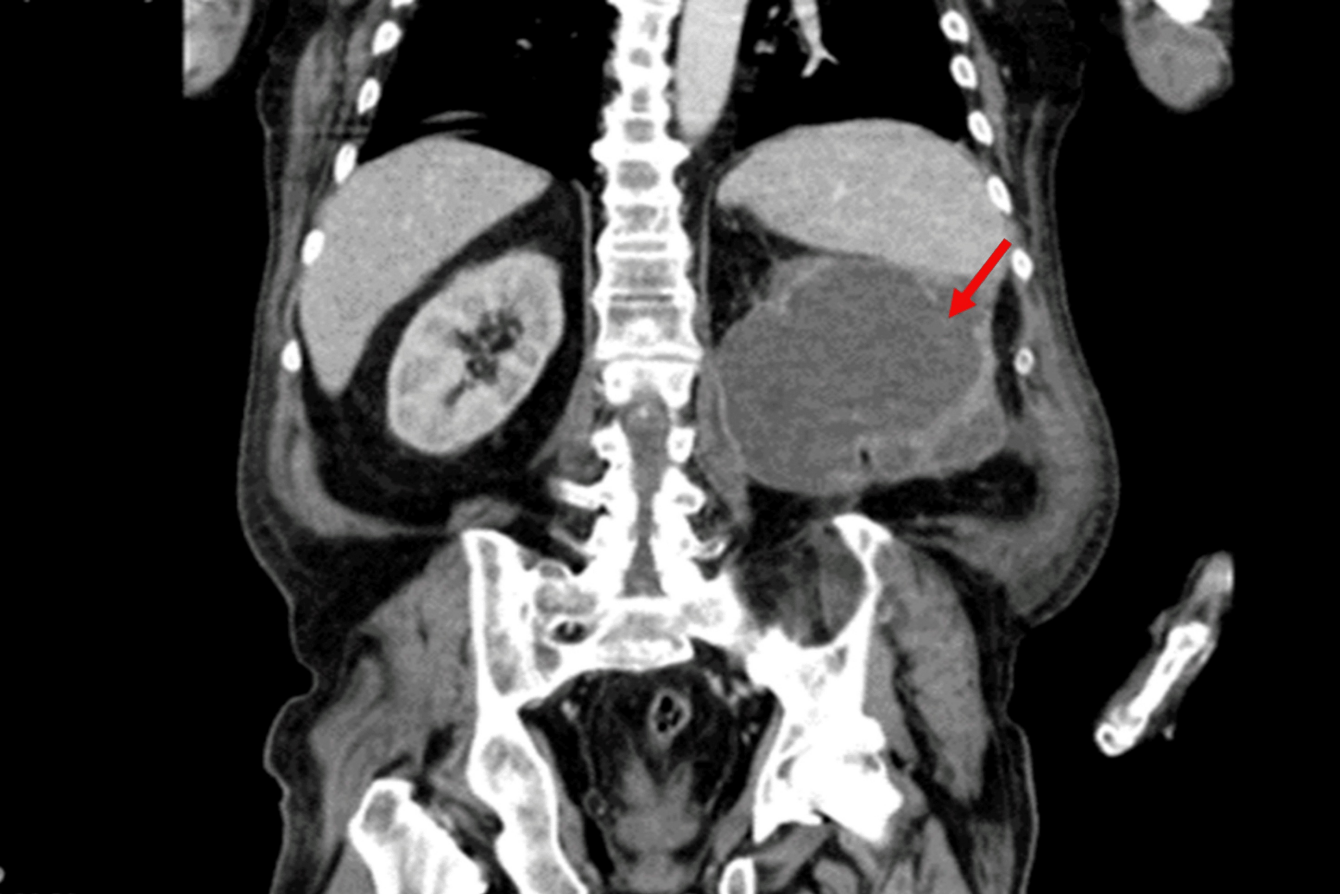
Abdominal CT scan at admission showing well-defined left cystic renal mass (13 cm) consistent with a cystic nephroma, associated with mild hydronephrosis and renal parenchymal atrophy

An abdominal computed tomography (CT) was performed, showing a well-defined left cystic renal mass (13 cm) consistent with a cystic nephroma, associated with mild hydronephrosis and renal parenchymal atrophy (Figure 1).

Blood and urine cultures were negative. *Mycobacterium tuberculosis* urine culture or polymerase chain reaction (PCR) wasn’t performed. During hospitalization, the patient underwent ultrasound-guided drainage and aspiration of the renal mass, after which a 12F pigtail catheter was placed for continuous drainage. The aspirated fluid was sent for cytological and microbiological analysis, including testing for *M. tuberculosis*. *Escherichia coli* and *M. tuberculosis* were both isolated from the sample (Table 2).

**Table 2:** Aspirated fluid analysis DNA: deoxyribonucleic acid

Aspirated fluid analysis	
Bacteriologic culture	Gram negative bacillus: *Escherichia coli*
Mycobacterial culture (acid-fast bacilli)	Positive
Detection of *Mycobacterium tuberculosis* complex DNA	Positive
Detection of rifampicin resistance	Negative
Cytological result	Proteinaceous material, cellular detritus, and rare histiocytes; consistent with cystic lesion content

**Figure 2:**
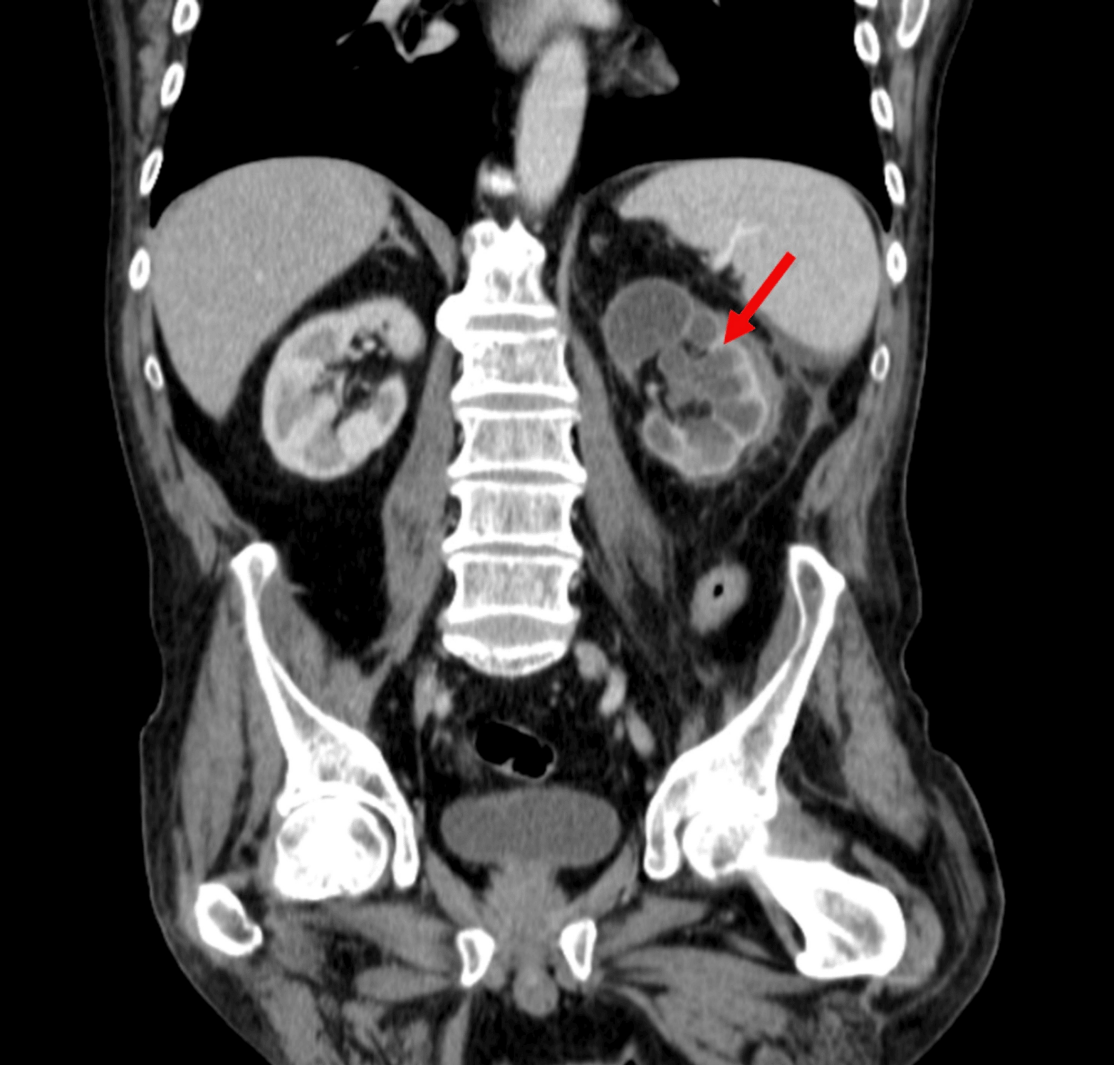
Control abdominal CT scan showing slight posterior left peri-renal densification, adjacent to the drain, with a slight reduction compared to the previous examination (measuring 47 x 26 mm).

The patient completed an antibiotic course of ceftriaxone, targeting the isolated *E. coli*, and initiated anti-tuberculous therapy with isoniazid, pyrazinamide, ethambutol, and rifampicin. Bronchofibroscopy was performed and pulmonary TB was excluded. The patient’s condition improved, and after two weeks, a follow-up abdominal CT scan showed a reduction in the cystic mass (4 cm) (Figure 2).

After 10 days, the pigtail catheter was removed. The patient's outcome was favorable, with preservation of apyrexia and negative inflammation markers (C reactive protein (CRP) 0.40 mg/dL). He was referred to the Pulmonary Diagnostic Centre to continue anti-tubercular therapy. 

## Discussion

Extrapulmonary TB, defined as *M. tuberculosis* infection occurring outside the pulmonary parenchyma, encompasses a broad spectrum of clinical manifestations and anatomical sites, with genitourinary involvement, particularly renal TB, constituting one of the most common forms [2,3]. Although pulmonary TB still dominates global incidence. Recent epidemiological trends highlight the increasing importance of extrapulmonary disease, which may represent up to 15-20% of all TB cases in immunocompetent patients and even higher proportions among the immunocompromised [1,6].

Renal TB typically arises from hematogenous dissemination of *M. tuberculosis* bacilli following either a primary infection or the reactivation of dormant foci [5]. The kidneys receive a substantial blood supply, allowing mycobacteria often seeded silently during initial infection to establish granulomatous lesions in the renal cortex. These lesions can remain quiescent for years before reactivation, often triggered by host immunocompromise. Progression may lead to the formation of caseating granulomas, cavitation, and strictures, ultimately causing parenchymal damage and obstructive complications [3,7].

Unlike pulmonary TB, which frequently presents with respiratory symptoms, renal TB often manifests insidiously. Patients may report nonspecific systemic symptoms such as low-grade fevers, malaise, weight loss, and night sweats or slight urinary signs such as sterile pyuria or microscopic hematuria [6,8,9]. The absence of pulmonary signs and the paucity of early urinary symptoms frequently delay diagnosis, leading clinicians to consider differential diagnoses such as neoplasms, complicated cystic renal disease, or other chronic infections.

Definitive diagnosis rests on a combination of imaging, histopathology, and microbiological testing. Imaging modalities such as ultrasound and CT may illustrate characteristic findings, such as calcifications, strictures, or cavitary lesions, but can also be nonspecific [3,7]. Direct evidence of *M. tuberculosis* via culture or PCR remains the gold standard, although molecular methods (e.g., Xpert® MTB/RIF; Cepheid, Sunnyvale, California, United States) have significantly improved diagnostic turnaround times and sensitivity, particularly in paucibacillary extrapulmonary settings [10].

The management of renal TB aligns closely with principles established for pulmonary disease. Standard multidrug anti-tuberculous therapy generally includes isoniazid, rifampicin, pyrazinamide, and ethambutol in the intensive phase, followed by a continuation phase of isoniazid and rifampicin. The preferred frequency of dosing for extrapulmonary TB is once daily for both phases [11]. Treatment duration may vary based on the severity and response, but a minimum of six months is typically recommended (Table 3) [3,6].

**Table 3:** Treatment regimen of renal TB EMB: ethambutol; INH: isoniazid; PZA: pyrazinamide; RIF: rifampicin; TB: tuberculosis

Intensive Phase		Continuation Phase	
Drug	Interval (minimum duration)	Drug	Interval (minimum duration)
INH, RIF, PZA, EMB	Daily for two months	INH, RIF	Daily for four months

Besides, surgical treatment as an adjunct to TB drugs is indicated for complications of urogenital TB. Nephrectomy is required for severely damaged kidneys, and reconstruction procedures include pyeloureteral anastomosis, ureterocalyceal anastomosis, caliceal reconstruction, uretero-ureteral anastomosis, and ureter substitution by ileum [3,6].

Drug-resistant TB is a concern and it can arise from spontaneous mutations, inadequate treatment regimens, poor adherence, or malabsorption, with risk factors including prior inadequate treatment, living in high-prevalence areas, and exposure to drug-resistant cases [1,11]. The WHO's 2022 guidelines introduce significant advancements in the treatment of multidrug-resistant TB (MDR-TB). A notable recommendation is the six-month all-oral BPaLM (bedaquiline, pretomanid, linezolid, and moxifloxacin) regimen for patients with MDR/rifampicin-resistant (RR) TB and those with additional fluoroquinolone resistance (pre-XDR-TB). This regimen offers improved outcomes and a shorter treatment duration, enhancing patient quality of life. Additionally, a nine-month all-oral regimen is recommended for MDR/RR-TB patients without fluoroquinolone resistance [12].

Renal TB requires careful follow-up due to the potential for relapse, often influenced by treatment duration and patient conditions. Recurrence rates can reach 19% after 12 months of therapy, particularly in regions with poor health conditions, highlighting the need for extended treatment durations and regular monitoring. Follow-up should include clinical evaluations, urinalysis, imaging, and bacteriological tests over at least five years to detect relapses early and manage complications like renal dysfunction or obstruction​ [13,14].

## Conclusions

This case highlights how renal TB can present subtly and be easily mistaken for other pathologies. The patient exhibited nonspecific symptoms (fatigue, weight loss, intermittent fever, and night sweats) without any pulmonary manifestations, and the initial imaging suggested a cystic renal lesion consistent with a cystic nephroma. The simultaneous finding of both *E. coli* and *M. tuberculosis* in the aspirated fluid underscores the complexity and rarity of this coinfection, compounded by the patient’s immunosuppressed status due to diabetes. Ultrasound-guided drainage was essential to confirm the diagnosis.

The importance of considering TB in the differential diagnosis of cystic renal lesions becomes evident when faced with persistent systemic symptoms and an unclear clinical picture. Although initial urine tests failed to reveal the pathogen, direct sampling from the lesion allowed for microbiological confirmation and guided appropriate treatment. Early recognition, targeted diagnostic methods, and specific treatment are critical to preventing permanent renal damage and systemic complications, particularly in immunocompromised patients.
